# Rhizospheric *Bacillus* spp. Rescues Plant Growth Under Salinity Stress *via* Regulating Gene Expression, Endogenous Hormones, and Antioxidant System of *Oryza sativa* L

**DOI:** 10.3389/fpls.2021.665590

**Published:** 2021-06-11

**Authors:** Muhammad Aaqil Khan, Muhammad Hamayun, Sajjad Asaf, Murtaza Khan, Byung-Wook Yun, Sang-Mo Kang, In-Jung Lee

**Affiliations:** ^1^Department of Applied Biosciences, Kyungpook National University, Daegu, South Korea; ^2^Department of Botany, Abdul Wali Khan University, Mardan, Pakistan; ^3^Natural and Medical Science Research Center, University of Nizwa, Nizwa, Oman

**Keywords:** salinity stress, growth attributes, hormones, antioxidants, genes expression, rice

## Abstract

Salinity has drastically reduced crop yields and harmed the global agricultural industry. We isolated 55 bacterial strains from plants inhabiting the coastal sand dunes of Pohang, Korea. A screening bioassay showed that 14 of the bacterial isolates secreted indole-3-acetic acid (IAA), 12 isolates were capable of exopolysaccharide (EPS) production and phosphate solubilization, and 10 isolates secreted siderophores. Based on our preliminary screening, 11 bacterial isolates were tested for salinity tolerance on Luria–Bertani (LB) media supplemented with 0, 50, 100, and 150 mM of NaCl. Three bacterial isolates, ALT11, ALT12, and ALT30, had the best tolerance against elevated NaCl levels and were selected for further study. Inoculation of the selected bacterial isolates significantly enhanced rice growth attributes, viz., shoot length (22.8–42.2%), root length (28.18–59%), fresh biomass (44.7–66.41%), dry biomass (85–90%), chlorophyll content (18.30–36.15%), Chl a (29.02–60.87%), Chl b (30.86–64.51%), and carotenoid content (26.86–70%), under elevated salt stress of 70 and 140 mM. Furthermore, a decrease in the endogenous abscisic acid (ABA) content (27.9–23%) and endogenous salicylic acid (SA) levels (11.70–69.19%) was observed in inoculated plants. Antioxidant analysis revealed an increase in total protein (TP) levels (42.57–68.26%), whereas it revealed a decrease in polyphenol peroxidase (PPO) (24.63–34.57%), glutathione (GSH) (25.53–24.91%), SOA (13.88–18.67%), and LPO levels (15.96–26.06%) of bacterial-inoculated plants. Moreover, an increase in catalase (CAT) (26–33.04%), peroxidase (POD) (59.55–78%), superoxide dismutase (SOD) (13.58–27.77%), and ascorbic peroxidase (APX) (5.76–22.74%) activity was observed. Additionally, inductively coupled plasma mass spectrometry (ICP-MS) analysis showed a decline in Na^+^ content (24.11 and 30.60%) and an increase in K^+^ (23.14 and 15.45%) and Mg^+^ (2.82 and 18.74%) under elevated salt stress. *OsNHX1* gene expression was downregulated (0.3 and 4.1-folds), whereas the gene expression of *OsPIN1A, OsCATA*, and *OsAPX1* was upregulated by a 7–17-fold in bacterial-inoculated rice plants. It was concluded that the selected bacterial isolates, ALT11, ALT12, and ALT30, mitigated the adverse effects of salt stress on rice growth and can be used as climate smart agricultural tools in ecofriendly agricultural practices.

## Introduction

Soil salinity is abiotic stress that significantly limits agricultural productivity and food security (Shrivastava and Kumar, 2015; Kumar et al., 2020). According to estimates, there is more than 830 million ha of salt-affected agricultural land globally. Of the 230 million ha of irrigated land worldwide, 45 million ha (20%) has been affected by high salt concentrations (Munns, 2005; Hoang et al., 2016). It is predicted that 50% of arable land will be threatened by 2050 due to soil salinization (Khan et al., 2019b,e; Shultana et al., 2020a). The salinization of agricultural lands occurs because of the accumulation of salts in the soil, particularly sodium and chloride ions, which leads to hypertonic stress (Kumar et al., 2020). High sodium (Na^+^) accumulation limits water conductance, disturbs the nutrient balance, and negatively impacts intercellular potassium (K^+^) influx, with K being an essential element required for plant growth (Kumar et al., 2020). Plants under salinity stress undergo several morphological changes, such as reduced seed germination, seedling growth and yield, and related physiological and molecular changes, which impede their growth and development. Additionally, high salinity stress causes oxidative stress and enhances the production of reactive oxygen species (ROS), such as singlet oxygen (^1^O_2_), superoxide anion (O^−2^), and hydrogen peroxide (H_2_O_2_). High ROS generation damages cell membranes, lipids, and nucleic acid, and leads to programmed cell death (Jha and Subramanian, 2013; Habib et al., 2016; Khan et al., 2018; Santos et al., 2018). To reduce the toxic effect of salinity, the plant antioxidant system, including enzymatic [superoxide dismutase (SOD), peroxidase (POD), and catalase (CAT)] and non-enzymatic [glutathione (GSH), total protein (TP)] antioxidants, has to be activated to control the biosynthesis of ROS and maintain them at a low level (Jha and Subramanian, 2013; Habib et al., 2016; Santos et al., 2018; Khan et al., 2020b). SOD is a metalloenzyme that protects cells from oxidative damage, and it catalyzes the conversion of superoxide radicals to H_2_O_2_, whereas ascorbic peroxidase (APX) and CAT break down H_2_O_2_ to H_2_O and O_2_ produced by SOD (Santos et al., 2000; Jaleel et al., 2009; Habib et al., 2016).

Plants respond to salinity stress in a complex manner involving hormonal regulation, gene expression, and signal pathways (Jamil et al., 2011). Phytohormones are small chemicals that play a crucial role in plant growth and development (Yu et al., 2020). During stress conditions, plant endogenous abscisic acid (ABA), and salicylic acid (SA) are produced as a part of a stress response (Khan et al., 2020c; Yu et al., 2020). ABA plays an irreplaceable role in plant tolerance and adaptation to a variety of stresses, and it accumulates in plants under stress, stimulating stomatal closure, adaptive physiological responses, and change in gene expression (Xiong et al., 2002; Kim et al., 2010; Li et al., 2010; Sah et al., 2016; Khan et al., 2020c; Yu et al., 2020). SA is an endogenous growth regulator phytohormone that induces stress tolerance in plants, including salinity stress (Sakhabutdinova et al., 2003; Ali et al., 2015; Yu et al., 2020). Besides participating in plant biotic stresses, SA plays an important role in plant salt tolerance by enhancing antioxidant systems, synthesizing osmolytes, and promoting photosynthesis and plant growth under salt stress (Filgueiras et al., 2019; Ahanger et al., 2020; Khan et al., 2020b; Yu et al., 2020). The regulation of genes is one of the key phenomena in plants by which they respond and try to adapt to salt stress (Jamil et al., 2011). In rice, *OsNHX1* is the most abundant Na^+^/H^+^ antiporter, and its gene expression is induced by different abiotic stresses, namely, high salinity and drought (Fukuda et al., 2004; Almeida et al., 2017). The phytohormone auxin regulates a variety of developmental processes in plant growth and root system architecture. A number of auxin influx genes, such as PIN and YUCCA, play a vital role in auxin biosynthesis, and they have been isolated, characterized, and can be exploited to help the plant adapt to various adverse environments, including high salinity environments (Khan et al., 2020a).

In the past three decades, diverse strategies, such as plant genetic engineering and molecular marker-assisted breeding approaches, have been used to develop saline-tolerant crops (Hamayun et al., 2017; Khan et al., 2019a, 2020a). However, these approaches are insufficient, labor-intensive, and time-consuming (Kumar et al., 2020). Currently, the use of plant growth-promoting bacteria (PGPB) to ameliorate abiotic stress is gaining importance in agricultural biotechnology and momentum for consideration (Nautiyal et al., 2013; Santos et al., 2018) by various direct and indirect mechanisms, such as the production of indole-3-acetic acid (IAA), exopolysaccharide (EPS) and organic acid siderophore, and phosphate solubilization (Nautiyal et al., 2013). These beneficial plant–microbe interactions are very frequent in nature and act as elicitors for tolerance to abiotic stress, including salinity stress (Nautiyal et al., 2013; Shultana et al., 2020a). Previously published reports showed that PGPB has been used for the mitigation of salinity stress in cucumber (Kang et al., 2014; Kartik et al., 2020), tomato (Kang et al., 2019), soybean (Khan et al., 2019b,c), rice (Nautiyal et al., 2013; Mukherjee et al., 2019) lettuce, and maize (Rojas-Tapias et al., 2012; Rafiq et al., 2020) plants.

Rice is a semiaquatic crop and the third most commonly valuable cereal crop after wheat and maize, feeding more than one-half of the world's population (Hoang et al., 2016). Rice is susceptible to salt stress when it is a seedling and during the early vegetative stage, and its productivity declines at low salt concentrations (Hoang et al., 2016; Tisarum et al., 2020). Hoang et al. (2016) reported that 10–50% yield loss was recorded at ECe 3.5–7.2 dSm^−1^. Several studies have emphasized the use of beneficial microorganisms, such as *Azospirillum, Bacillus, Rhizobium, Serratia*, and *Pseudomonas*, which can tolerate salinity stress and enhance the growth and productivity of crop plants (Nautiyal et al., 2013; Kang et al., 2014, 2019; Santos et al., 2018; Khan et al., 2019a,b). Therefore, we hypothesized that halotolerant microbes with multiple plant growth-promoting traits could be used in climate smart agriculture to enhance plant tolerance against salinity stress. Thus, we isolated bacteria and screened them for different plant growth promotion (PGP) traits, including IAA, siderophore production, EPS production, tolerance to NaCl stress, and identified isolates ALT11, ALT12, and ALT30 based on halotolerance and multiple PGP traits. The objective of this study was to understand the effect of selected isolates upon inoculation on the growth and chlorophyll content of rice under normal conditions and salinity stress of 70 and 140 mM. Furthermore, to elucidate the translocation of Na^+^, K^+^, and magnesium (Mg^+^) uptake, the production of various antioxidant (SOD, CAT, POD, APX) and non-enzymatic antioxidants [GSH, LPO, TP, polyphenol peroxidase (PPO)] was determined. Moreover, the expression pattern of salt-related genes under salinity stress in rice plants inoculated with or without selected bacterial isolates was investigated.

## Materials and Methods

### General Procedure

Plant roots with adhered soil were collected from *Artemisia princeps, Chenopodium ficifolium, Oenothera biennis*, and *Echinochloa crus-galli* growing in the sand dunes at Pohang beach. For bacterial isolation, root samples were preserved inside an icebox, and subsequent analyses were undertaken at the Crop Physiology Laboratory, Department of Applied Biosciences, Kyungpook National University, Korea. Plant roots with adhered soil were transferred into a conical flask (99 mL sterilized distilled water and shaken for 3 min). The samples were diluted through a series of 1-fold dilutions, and 0.1 mL of solution was spread on LB agar plates and incubated at 28°C. Bacterial colonies were collected based on their colony morphology, and they were preserved in 75% glycerol stock until further use.

### Screening of Bacterial Isolates for Their Plant Growth-Promoting Capacity

Bacterial isolates were screened for different PGP characteristics, including IAA, siderophore production, phosphate solubilization, and organic acid and EPS production. For IAA production, a Salkowski reagent was used by adding 1 mL of supernatant and 1 mL of Salkowski reagent for 30 min in the dark. The development of a pink color indicated IAA production (Kang et al., 2020). Trypticase soy agar medium supplemented with Ca_3_(PO_4_)_2_ was used for phosphate solubilization, and the plates were incubated at 30°C for 7 days, and the formation of transparent halos around each colony was observed (Kubi et al., 2021). For siderophore production, chromeazurol “S” agar plates were incubated at 30°C until the appearance of orange halos, which contrasted with the blue background (Khan et al., 2020a). However, for determining bacterial EPS production, a Congo red assay [LB broth (25 g/L, sucrose (5%), Congo red (0.8 g/L)] was incubated for 4 days at 30°C (Kim et al., 2020).

### Molecular Identification of Selected Bacterial Isolates

The selected bacterial strains were identified using the 16S rRNA gene through PCR using universal primer [27F primer (5′-AGAGTTTGATC (AC) TGGCT CAG-3′) and 1492R primer (5′-CGG(CT)TACCTTGTTA CGACTT-3′)]. BLASTn searching of EzTaxon and NCBI based on PCR amplification and sequencing of the 16S rRNA gene region was conducted to identify these bacterial strains at the molecular level. The BLASTn search identified isolates with 100% homology to their respective species. Additionally, the maximum-likelihood (ML) method was used to construct a phylogenetic tree for 16S with MEGA 10 after sequence alignment using Clustal W (version 7.222). To obtain a consensus tree and confirm the molecular identification of these strains, the respective sequences of the 16S rRNA gene region showing similarity to our endophytic isolates were aligned at 1,000 bootstrap replications.

### Indole-3-Acetic Acid and Organic Acid Production by Bacterial Isolates

The selected bacterial isolates were grown in LB media (10 g tryptone, 5 g yeast extract, pH 7.2) and centrifuged (5,000 × g; 10 min). The culture filtrates were centrifuged after 3 days to separate cells into a free culture, which were further acidified to a pH of 2.8 and supplemented with 40 μL (D5)-IAA as an IAA internal standard by following the standard protocol (Jan et al., 2019). The acidified and standard supplemented free culture cells were extracted, methylated, and injected into a GC/MS-SIM (6890N network GC system and 5,973 network mass selective detector; Agilent Technologies, the United States) for the identification and quantification of IAA (Sahile et al., 2021). For organic acid analysis, the cultural broth was filtered [0.22 um SmartPor Syrange Filter (P/N SPU0213-1)] and 10 ul was used to conduct high-performance liquid chromatography (HPLC; Waters 600, Milford, MA, the United States). The retention time and peak area were compared using the standard from Sigma-Aldrich, the United States (Khan et al., 2020c).

### Bioassay for Growth Promotion Under Salt Stress

Seeds of rice (*Oryza sativa* L. var. Jin-so-mi) were surface-sterilized and incubated, and uniformly germinated seedlings were transferred to sterilized pots filled with autoclaved rice paddy purchased from Korea (Adhikari et al., 2020). The entire experiment was conducted in a growth chamber: day/night cycle 14 h at 30°C/10 h at 25°C; relative humidity 60–70%; light intensity 250 μmol/m^−2^s^−1^. The experiments consisted of (A) control rice plants (Control; only distilled water); (B) rice plants inoculated with ALT11, ALT12, and ALT30; (C) 70 mM NaCl stress; (D) 70 mM NaCl stress with the inoculation of isolates ALT11, ALT12, and ALT30; (E) 140 mM NaCl stress; and (F) 140 mM NaCl stress with the inoculation of isolates ALT11, ALT12, and ALT30. Bacterial culture (grown in LB media) was kept in a 250-ml bottle and centrifuged at 6,000 × g (10 min; 4°C). The pellets obtained were diluted with sterilized distilled water. Each pot was inoculated with 50 ml of freshly diluted bacterial culture (10^8^ CFU), and this inoculation was repeated another three times after a further 5 days. After 15 days, 80 ml of salt solution (70 and 140 mM NaCl) was given to each pot for 7 days. Two weeks after the salt stress treatment, the plants were harvested and morphological parameters, such as root length, shoot length, fresh and dry biomass, and chlorophyll content (SPAD-502, Konica Minolta, Japan), were measured. The leaf chlorophyll (*Chl a* & *Chl b*) and carotenoid contents were determined following the standard protocol (Khan et al., 2020c, 2021). The absorbance readings of the *Chl a, Chl b*, and carotenoid contents were taken at wavelengths of 663, 645, and 580 nm using a spectrophotometer.

Chlorophyll a (mg/g FW) = [{(12.7^*^A_663_) – (2.69^*^A_645_)} 1000^*^W]^*^V

Chlorophyll b (mg/g FW) = [{(22.9^*^A_645_) – (4.68^*^A_663_)} / 1000^*^W]^*^V

Carotenoids (μg/g FW) = A_480_+(0.638^*^A_663_) – (0.638^*^A_645_) Chlorophyll a, b and carotenoid contents were calculated using the following formulae:

where A+ Absorbance at respective wave length; W = fresh weight and V = extraction volume.

Each treatment was replicated three times, and each replica has five pots having one plant in each pot. The whole experiment was independently repeated three times.

### Estimation of Enzymatic and Non-enzymatic Antioxidant Activities

For antioxidant analysis, leaves were homogenized in a 50 mM phosphate buffer (pH 7.5) containing 1.0% (w/v) polyvinylpyrrolidone (PVP), 0.1 mM EDTA, and 0.5% (w/v) Triton X-100. SOD activities were measured spectrophotometrically (Multiskan GO; Thermo Fisher Scientific, Waltham, MA, the United States) at 560 nm following the procedure described in the study by Marklund and Marklund (1974). GSH was measured following the procedure described in the study by Asaf et al. (2017a). Briefly, 0.2 g samples were ground and homogenized in 3 mL of 5% trichloroacetic acid (TAC). Then, they were centrifuged, the supernatant (0.1 mL) was mixed with 150 mM monosodium phosphate buffer (3 mL) and Ellman's reagent (0.5 mL), and this was measured spectrophotometrically at 412 nm. CAT activity was determined following the procedure described in the study by Radhakrishnan and Lee (2013), and the resulting absorbance was measured at a wavelength of 240 nm. Similarly, PPO was estimated using the same reaction mixture of POD excluding H_2_O_2_, and the resulting reaction was measured at 420 nm wavelength (Khan et al., 2019e). Additionally, superoxide anions (SOAs) were measured following the procedure described in the study by Khan et al. (2018) and Khan et al. (2019d) at 580 nm, lipid peroxidation was assayed following the procedure described in the study by Khan et al. (2020b) measured at 532 nm wavelength, and leaf APX activity was estimated at 290 nm following the procedure described in the study by Kang et al. (2019).

### Quantification of Endogenous Phytohormones Under Salt Stress

The endogenous phytohormones ABA and SA were quantified. For SA analysis, 0.2 g of freeze-dried sample was used with HPLC, and SA was quantified using fluorescence detection (Jan et al., 2019). For ABA analysis, 3 mg of powdered sample was mixed with 30 ml of extraction solution and 10 ng of ABA standard ([±]-3,5,5,7,7,7-d^6^). Furthermore, the extraction was dried and methylated with diazomethane for GC-MS/SIM analysis. The monitor responses to ions at *m*/*z* of 190 and 162 for Me-ABA, and 194 and 166 for Me-(^2^H_6_)-ABA were obtained using Lab-Base (ThermoQuest, Manchester, the United Kingdom) data system software (Asaf et al., 2017b).

### Inductively Coupled Plasma Mass Spectrometry Analysis

The Na^+^, K^+^, and Mg^+^ contents in rice plants inoculated with selected bacterial isolates were quantified using inductively coupled plasma mass spectrometry (ICP-MS). Lyophilized powder (0.2 g) samples were soaked in HCl (0.5 M) and oven-dried. The obtained digested samples were then analyzed using ICP-MS (Optima 7900DV; PerkinElmer, Waltham, MA, the United States) (Khan et al., 2020b).

### Estimation of Gene Expression Through RT-PCR

Quantitative real-time PCR (qRT-PCR) was analyzed as described earlier by Shahid et al. (2019). Total RNA was extracted using a TRIzol reagent, while a DiaStar™ RT kit (SolGent, Korea) was used for extracting cDNA. RT-PCR was performed in the EcoTM real-time PCR machine using 2X Real-time PCR Master Mix (including SYBR® Green I BioFACT™ Korea), using synthesized cDNA and gene-specific primers (Supplementary Table 1). To normalize the level of relative expression of each gene, actin was used for each reaction and the expression level was calculated in control plants relatively with other NaCl-inoculated and non-inoculated rice-stressed plants.

### Statistical Analysis

Data were collected in triplicate and subjected to the Duncan multiple range test using the SAS version 9.2 software. Furthermore, the data were graphically presented using the GraphPad Prism software (version 6.01, San Diego, CA, the United States).

## Results

### Isolation, Screening Bioassay, and Identification of Selected Bacterial Isolates

A total of 55 rhizospheric bacterial isolates were isolated and screened for different PGP traits, such as siderophore production, phosphate solubilization, EPS formation, and IAA production (Supplementary Table 2, Supplementary Figure 1). The screening results indicated that 20 isolates showed IAA activity. Fourteen isolates showed positive results for siderophore, whereas 12 isolates showed phosphate solubilization and EPS activity (Supplementary Figure 1). Based on multiple PGP traits, 11 isolates were screened for salt tolerance at three different concentrations of NaCl (50, 100, and 150 mM). Isolates ALT 11, ALT 12, and ALT 30 showed the highest tolerance of NaCl (Supplementary Figure 2). Therefore, these isolates were selected for further investigation using molecular identification. Our results revealed that rhizospheric bacteria ALT 11 showed sequence similarity with *Bacillus*. Additionally, the neighbor-joining method was employed to construct phylogenetic trees using 16S and MEGA 6 software after performing sequence alignment using Clustal W and a default parameter. The results revealed that isolates ALT11, ALT12, and ALT30 exhibited a high level of 16S rRNA sequence identity with *Bacillus*. Furthermore, these sequences were submitted to NCBI with accession number MW513461 for ALT11, MW513462 for ALT12, and MW513463 for ALT30 (Supplementary Figure 3).

### *In vitro* Indole-3-acetic Acid and Organic Acid of Isolate

**Figure 1:**
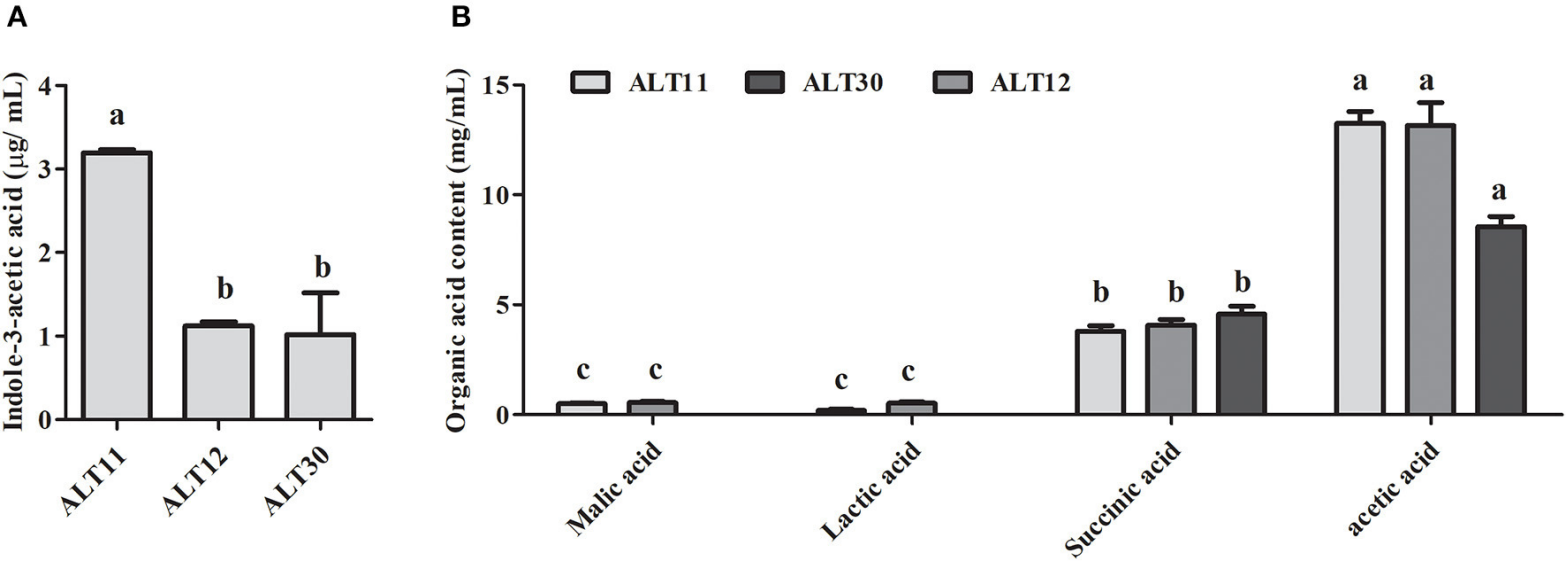
Quantification of indole-3-acetic acid (IAA) and organic acids produced by isolates ALT11, ALT12, and ALT30. **(A)** GC/MS-SIM analysis of the IAA content in the culture broth of isolate ALT1, **(B)** organic acid detection and quantification using high-performance liquid chromatography relative to their respective standard. Different letters indicate significant differences between the mean values of three replicates ± standard deviation.

The identified rhizospheric bacterial strain was grown in LB medium for 5 days, and the culture filtrate was tested for IAA, which was determined using GC-MS/SIM, and organic acid using HPLC. The IAA results showed that ALT11 produced the highest amount (3.5 μg/mL) followed by ALT12 and ALT30 (Figure 1A). Furthermore, the organic acid results showed that isolates ALT11 and ALT12 produced, in addition to succinic and acetic acid, malic and lactic acids (Figure 1B). Although acetic and succinic acids were produced by all isolates, the highest amounts of succinic acid were observed in the CF of isolate ALT30, whereas acetic acid was highly produced by ALT11 and ALT12 (Figure 1B).

### Bacterial Isolates Regulate Rice Growth Under Salinity Stress

**Figure 2:**
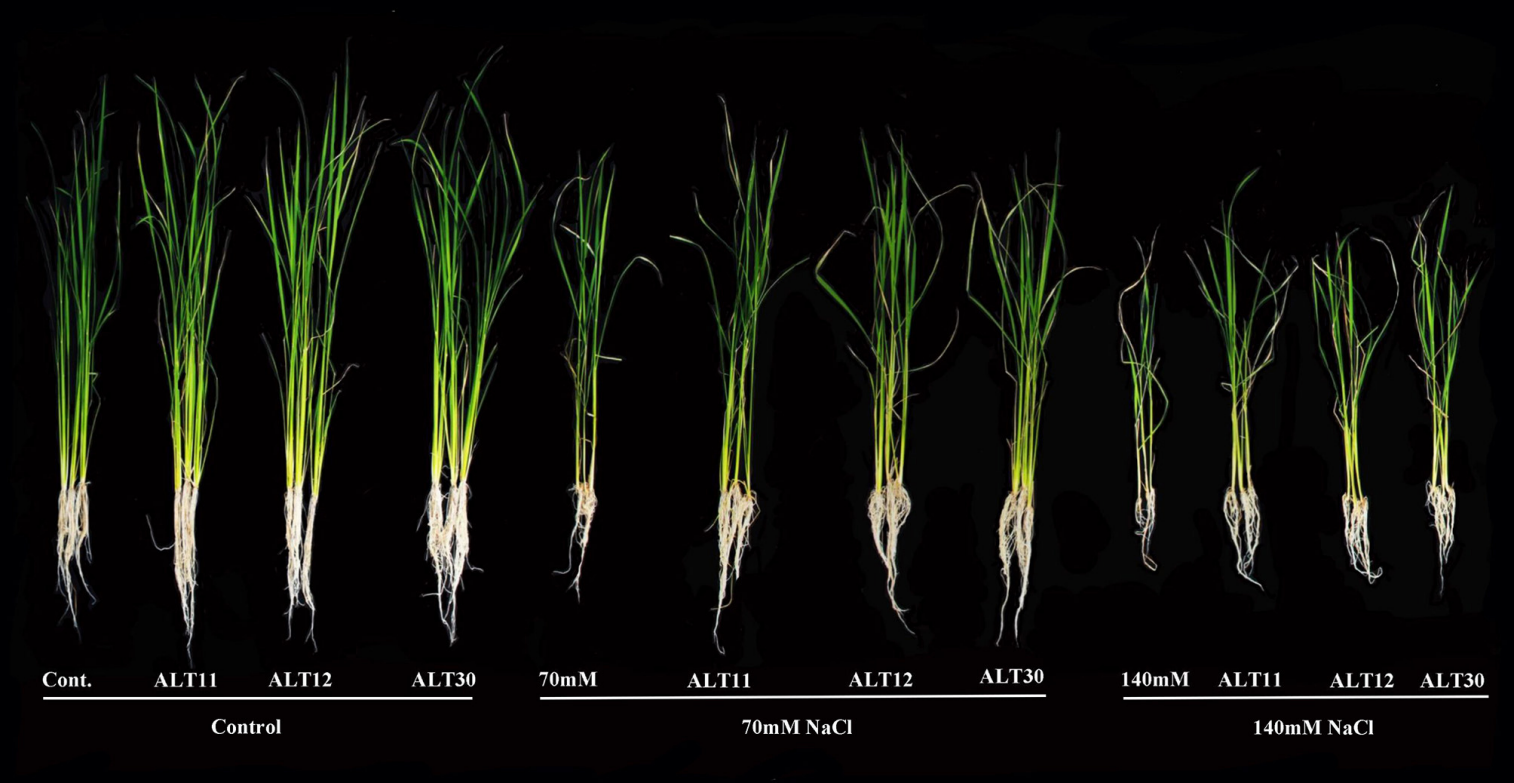
Effects of selected bacterial isolates, ALT11, ALT12, and ALT30, on the growth of rice plants (9 weeks old) under standard salinity and 70 and 140 mM NaCl stress.

Our results showed that increasing salinity stress adversely affected the growth attributes of rice plants (Figure 2). Under salinity stress, significant decreases were observed in shoot length (22.8–42.2%), root length (28.18–59%), fresh weight (44.7–66.41%), and dry weight (85–90%) in the 70 and 140 mM NaCl treatments compared with the rice plants in the control treatment (Table 1). The first value between parentheses shows 70 mM, while the second value shows 140 mM of NaCl stress. However, the plant–microbe interaction reverted the effect of salinity stress and caused increases in shoot length (27.03–46.5%), root length (29.11–40.81%), fresh weight (45.94–53.33%), and dry weight (59.42–66.66%) in bacterial-inoculated salt-stressed plants compared with the plants treated with 70 and 140 mM NaCl (Table 1). Similarly, increases in salinity (70 and 140 mM) also reduced the chlorophyll content (18.30–36.15%), *Chl a* (29.02–60.87%), *Chl b* (30.86–64.51%), and total carotenoid content (21.63–62.28%) decreased compared with the control plants, whereas there were increases in chlorophyll content (35.87–47.76%), *Chl a* (22.41–59.93%), *Chl b* (27.28–52.89%), and total carotenoid content (26.86–70%) in the bacterial-inoculated salt-stressed plants compared with the plants only treated with NaCl (70 and 140 mM; Table 2). Under normal conditions, increases in growth characteristics, such as shoot/root length (17.99/27.28%), fresh/dry weight (29.10/93.54%), chlorophyll content (23.92%), *Chl a* (14.74%), *Chl b* (7.03%), and total carotenoid content (16.95%), were observed in bacterial-inoculated rice plants compared with the control rice plants (Tables 1, 2).

**Table 1:** Growth-promoting effect of isolates ALT11, ALT12, and ALT30 on rice growth under normal conditions and 70 and 140 mM NaCl stress.

	**Shoot length** ** (cm)**	**Root length** ** (cm)**	**Fresh weight** ** (3 Plant/g)**	**Dry weight** ** (3 plant/g)**
**Plants without stress**				
Control	35.0 ± 2.0^b^	11.0 ± 0.3^c^	13.4 ± 0.5^b^	4.8 ± 0.1^b^
Isolate ALT11	41.3 ± 2.5^a^	14.0 ± 0.5^a^	17.2 ± 1.1^a^	6.2 ± 0.4^a^
Isolate ALT12	40.6 ± 1.5^a^	13.0 ± 0.3^b^	17.3 ± 0.7^a^	6.5 ± 0.5^a^
Isolate ALT30	39.5 ± 1.3^a^	13.1 ± 0.3b	17.0 ± 1.0^a^	6.3 ± 0.7^a^
**70 and 140 mM NaCl stress**				
70 mM NaCl	27.0 ± 1.0^d,e^	7.9 ± 0.4^e^	7.4 ± 0.5^d^	0.69 ± 0.1^c,d^
70 mM+ALT1	32.0 ± 2.0^c^	10.1 ± 0.3^d^	10.0 ± 1.0^c^	1.1 ± 0.1^c^
70 mM+ALT12	34.3 ± 1.5^b,c^	10.2 ± 0.2^d^	10.4 ± 1.0^c^	1.1 ± 0.1^c^
70 mM+ALT30	32.3 ± 2.5^b,c^	10.1 ± 0.3^d^	10.8 ± 0.8^c^	1.1 ± 0.2^c^
**140 mM NaCl stress**				
140 mM NaCl	20.0 ± 1.0^g^	4.9 ± 0.3^g^	4.5 ± 0.5^f^	0.30 ± 0.05^e^
140 mM+ALT1	25.0 ± 1.0^f^	6.9 ± 0.4^f^	6.4 ± 0.5^e^	0.5 ± 0.02^d^
140 mM+ALT12	29.3 ± 0.5^d^	6.9 ± 0.3^f^	6.9 ± 1.0^e^	0.50 ± 0.01^d^
140 mM+ALT30	28 ± 1.0^d^	7.8 ± 0.5^e^	5.9 ± 0.3^ef^	0.47 ± 0.02^d^

*Different letters indicate significant differences between the mean values of three replicates ± standard deviation*.

**Table 2:** The effect of NaCl stress on different chlorophyll contents of rice with and without the inoculation of isolates ALT11, ALT12, and ALT30.

	**SPAD**	***Chl a***	***Chl b***	**Carotenoid**
**Plants without stress**				
Control	24.53 ± 0.5^c^	36.14 ± 1.0^c^	68.19 ± 2.7^a^	3.42 ± 0.2^b^
Isolate ALT11	28.5 ± 1.0^b^	38.85 ± 1.0^b^	69.87 ± 2.0^a^	4.01 ± 0.7^a^
Isolate ALT12	30.40 ± 0.5^a^	41.47 ± 1.5^a^	72.99 ± 2.0^a^	3.98 ± 0.7^a^
Isolate ALT30	30.16 ± 0.7^a^	40.55 ± 1.5^a,b^	71.76 ± 1.5^a^	3.98 ± 0.2^a^
**70 mM NaCl stress**				
70 mM NaCl	20.04 ± 1.5^e^	25.65 ± 0.5^e^	47.14 ± 2.0^d^	2.68 ± 0.1^c^
70 mM+ALT11	23.07 ± 1.0^c,d^	29.57 ± 0.5^d^	53.89 ± 3.5^c^	3.19 ± 0.1^b^
70 mM+ALT12	27.23 ± 1.0^b^	30.06 ± 1.0^d^	60.06 ± 3.1^b^	3.46 ± 0.2^b^
70 mM+ALT30	22.21 ± 1.0^d^	31.45 ± 1.7^d^	50.78 ± 3.2^cd^	3.40 ± 0.1^b^
**140 mM NaCl stress**				
140 mM NaCl	15.66 ± 0.5^f^	14.14 ± 0.7^g^	24.20 ± 2.0^g^	1.29 ± 0.2^f^
140 mM+ALT11	19.63 ± 0.5^e^	20.31 ± 1.1^f^	32.97 ± 2.9^f^	1.73 ± 0.1^e^
140 mM+ALT12	18.94 ± 0.9^e^	22.19 ± 1.3^f^	37.79 ± 4.0^e^	2.20 ± 0.2d
140 mM+ALT30	23.14 ± 1.0^cd^	20.25 ± 1.0^f^	30.25 ± 1.98^f^	2.20 ± 0.2^d^

*A: Chlorophyll content (SPAD), chlorophyll A (Chl a), chlorophyll B (Chl b), and carotenoid contents. Different letters indicate significant differences between the mean values of three replicates ± standard deviation*.

### Bacterial Isolates Regulate Rice Endogenous Phytohormones

**Figure 3:**
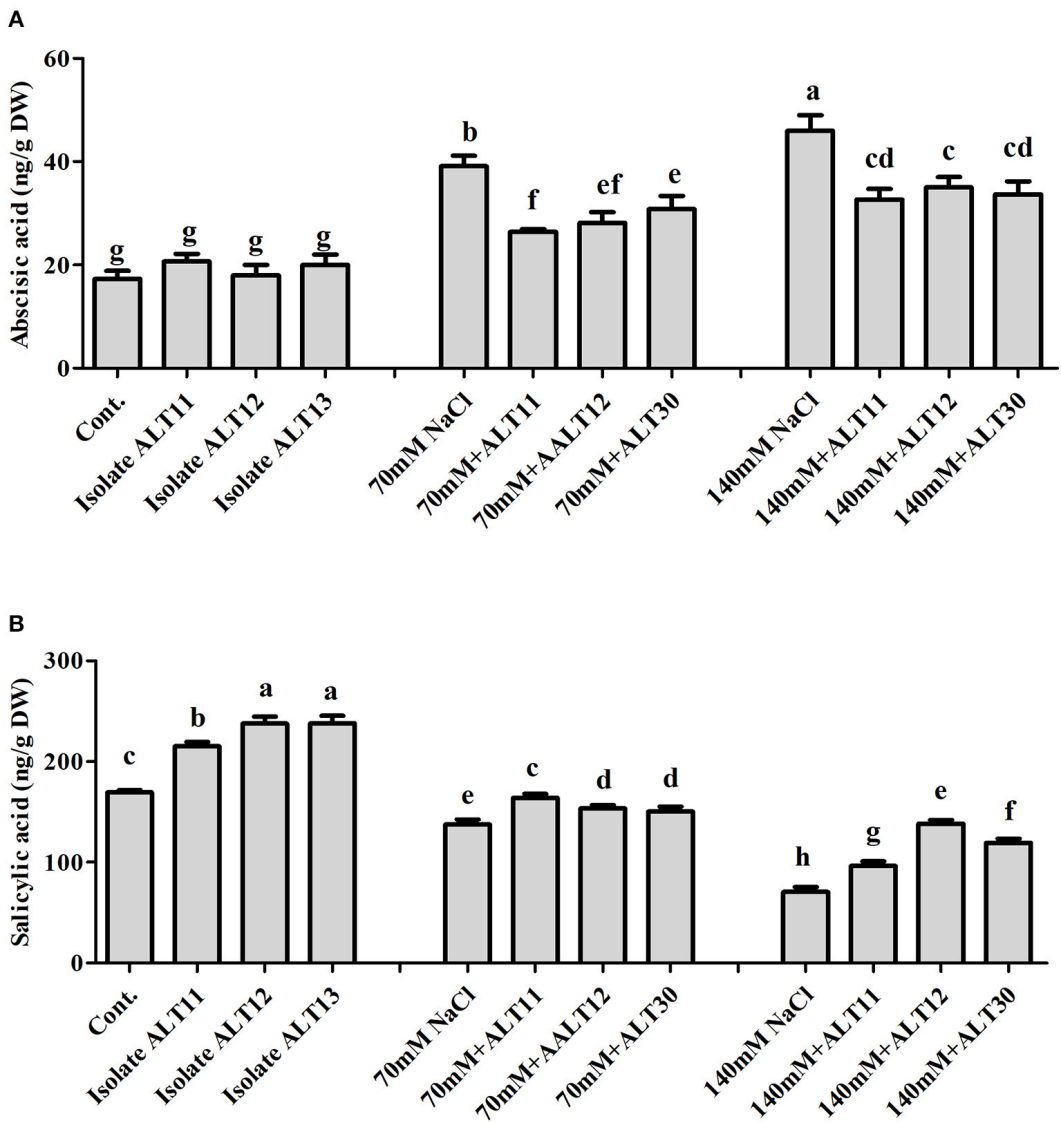
Endogenous abscisic acid (ABA) and salicylic acid (SA) quantification in rice plants inoculated with isolates ALT11, ALT12, and ALT30 under standard salinity and NaCl stress (70 and 140 mM). **(A)** Demonstrates ABA and **(B)** shows the amount of SA under normal conditions and NaCl stress. Different letters indicate significant differences between the mean values of three replicates ± standard deviation.

The endogenous ABA and SA contents were investigated in both inoculated and non-inoculated rice plants under normal and NaCl stress (70 and 140 mM). The ABA results showed a significant increase in ABA content under salinity stress (125–165.43%). However, rice plants inoculated with all three strains showed a significant decrease in ABA content (27.9–23%) compared with rice plants only treated with NaCl (Figure 3A). The SA content showed an opposite response: A decrease in endogenous SA content was observed under 70 mM (18%) and 140 mM (58.32%) NaCl-stressed rice plants, whereas there was an increase in the SA content (11.70–69.19%) in bacterial-inoculated salt-stressed rice plants compared with plants only treated with NaCl (Figure 3B).

### Regulation of Enzymatic and Non-enzymatic Antioxidants Under Salinity Stress

**Figure 4:**
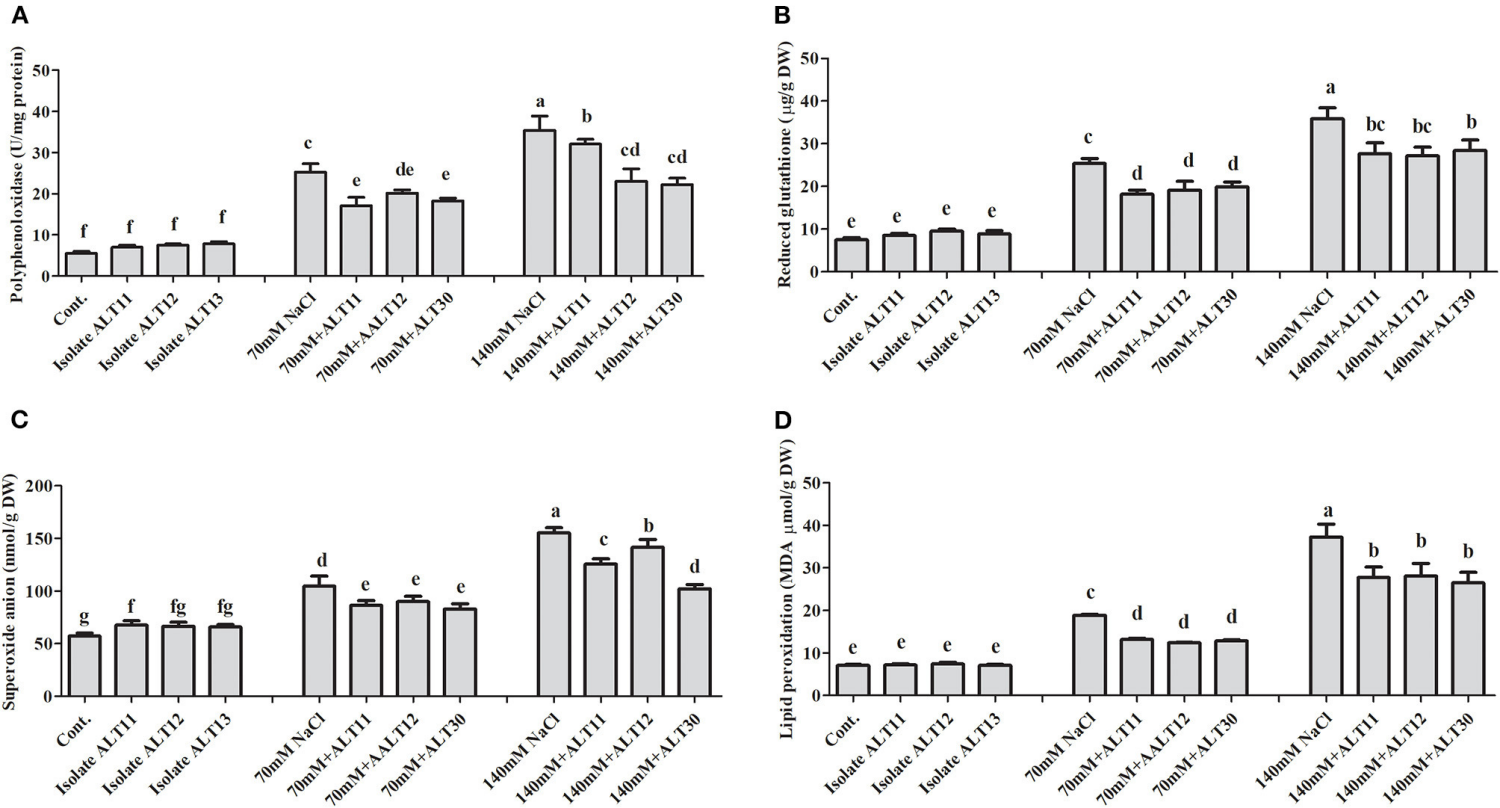
The effect of isolates ALT11, ALT12, and ALT30 on different antioxidants under normal conditions and NaCl stress (70 and 140 mM). **(A)** Polyphenol oxidase (PPO); **(B)** reduced glutathione (GSH); **(C)** superoxide anions (SOAs); **(D)** lipid peroxidation (MDA). Different letters indicate significant differences between the mean values of the three replicates ± standard deviation.

**Figure 5:**
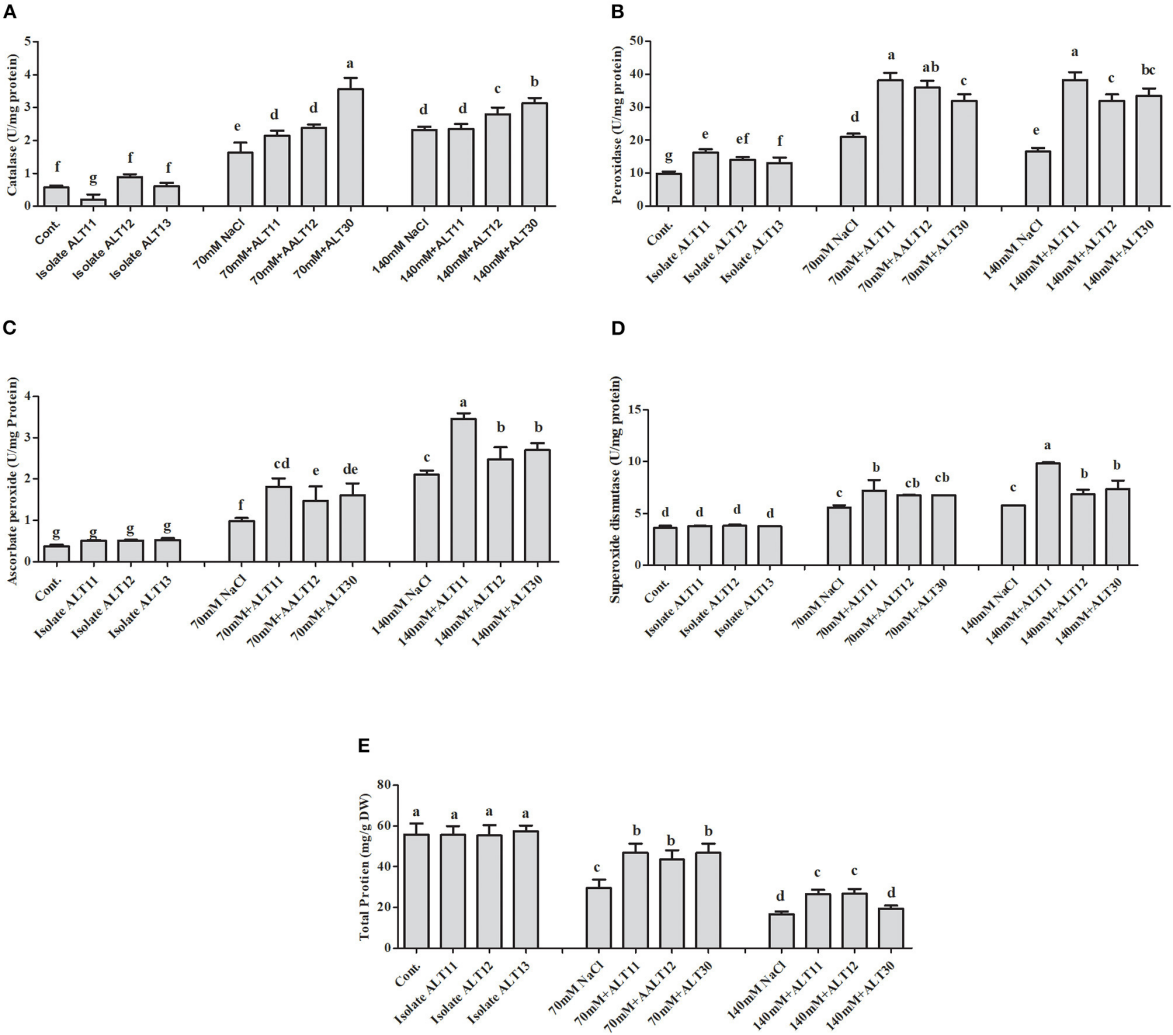
The effect of isolates ALT11, ALT12, and ALT30 on different antioxidants under normal conditions and NaCl stress (70 and 140 mM). **(A)** Catalase (CAT); **(B)** peroxidase (POD); **(C)** ascorbic peroxidase (APX); **(D)** superoxide dismutase (SOD); and **(E)** total protein (TP). Different letters indicate significant differences between the mean values of three replicates ± standard deviation.

Different enzymatic and non-enzymatic antioxidant activities were determined in rice plants under NaCl stress and bacterial-inoculated plants. CAT, POD, PPO, GSH, SOA, and APX activities were increased with increasing salinity stress, whereas the opposite trend was observed for the TP content (Figures 4, 5). However, bacterial-inoculated rice plants showed an increase in TP content and a decrease in PPO, GSH, SOA, and LPO. Under salinity stress (70 and 140 mM), there were significant increases in PPO (3.7–5.4-fold), GSH (2.5–4-fold), SOA (0.8–1.7-fold), and LPO (1–4.3-fold; Figures 4A–D). On the other hand, rice plants inoculated with isolate ALT showed a significant decrease in PPO (24.63–34.57%), GSH (25.53–24.91%), SOA (13.88–18.67%), and LPO (15.96–26.06%) compared with plants treated with only 70 and 140 mM NaCl (Figures 4A–D). However, an increase in CAT (1.6–2.3-fold), POD (1–1.7-fold), SOD (41.89–60.89%), and APX (1.6–4.4-fold) was observed (Figures 5A–D). There were significant increases in CAT (26–33.04%), POD (59.55–78%), SOD (13.58–27.77%), and APX (5.76–22.74%) in bacterial-inoculated rice plants compared with salt-stressed plants (Figures 5A–D). The TP content showed the opposite response, and a decrease in TP content (45.23–70.31%) was observed in 70 and 140 mM NaCl-stressed rice plants, while there was an increase in the TP content (42.57–68.26%) in bacterial-inoculated salt-stressed plants compared with plants treated with NaCl (Figure 5E).

### Inductively Coupled Plasma Mass Spectrometric Analysis of Na^+^, K^+^, and Mg^+^ Ions Under Salt Stress

**Figure 6:**
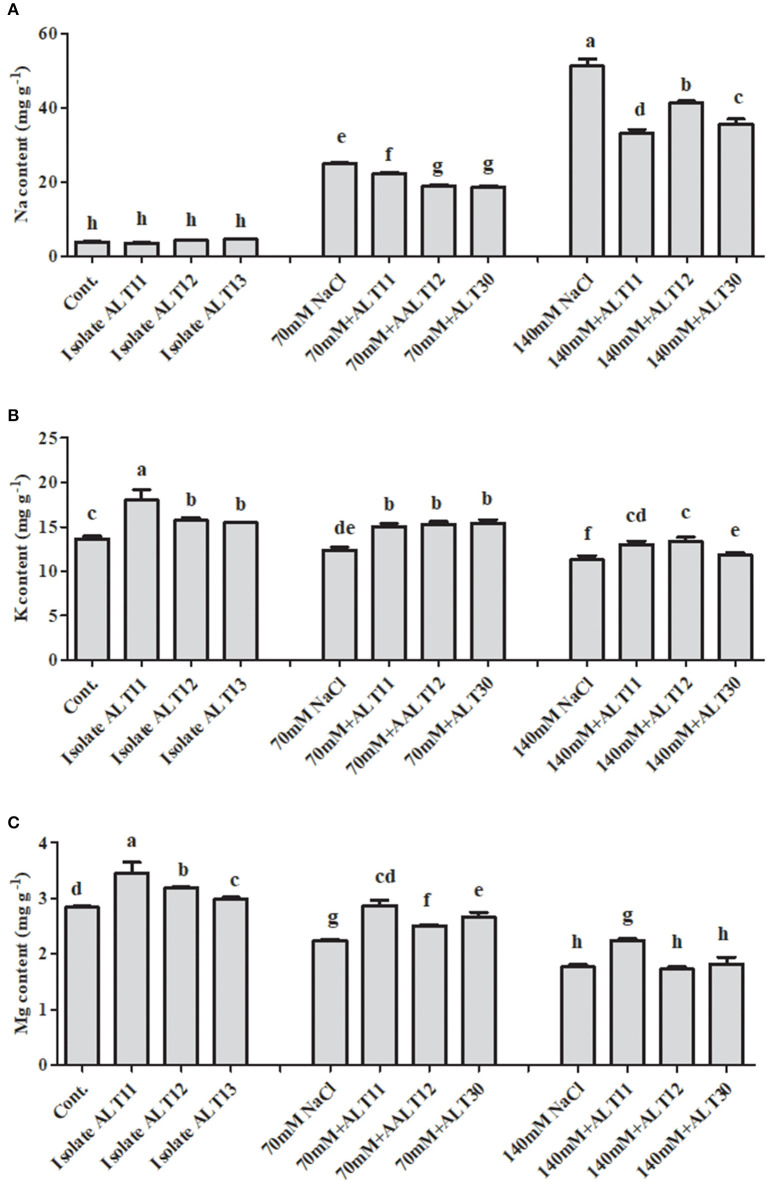
The effect of isolates ALT11, ALT12, and ALT30 on sodium (Na^+^), potassium (K^+^) and magnesium (Mg) contents under normal conditions and NaCl stress (70 and 140 mM). **(A)** Na^+^ content, **(B)** K^+^ content, and **(C)** Mg content in rice plants under normal conditions and NaCl stress. Different letters indicate significant differences between the mean values of three replicates ± standard deviation.

The ICP-MS results demonstrated that Na^**+**^, K^**+**^, and Mg^**+**^ accumulations were different in control, NaCl-stressed (70 and 140 mM), and bacterial-inoculated rice plants (Figure 6). The accumulation of Na increased at 70 mM (5.4-fold) and 140 mM (12.2-fold) in the NaCl-stressed rice plants compared with the control rice plants. However, there was a decrease in the Na content (24.11 and 30.60%) of rice plants inoculated with bacterial isolates compared with plants treated with only NaCl (Figure 6A). Additionally, as salinity increased (to 70 and 140 mM), the K (9.22 and 17.42%, respectively) and Mg (21.12 and 37.67%, respectively) content decreased (Figures 6B,C). However, increases in K (23.14 and 15.45%) and Mg (2.82 and 18.74%) contents were observed in bacterial-inoculated salt-stressed plants compared with plants only treated with 70 or 140 mM of NaCl (Figures 6B,C). Under normal conditions, in bacterial-inoculated rice plants, there is an increase in K (15.30%) and Mg (12.32%) contents, while no significant difference in Na (9.53%) content was observed compared with the control rice plants (Figures 6A–C).

### Estimation of Gene Expression

**Figure 7:**
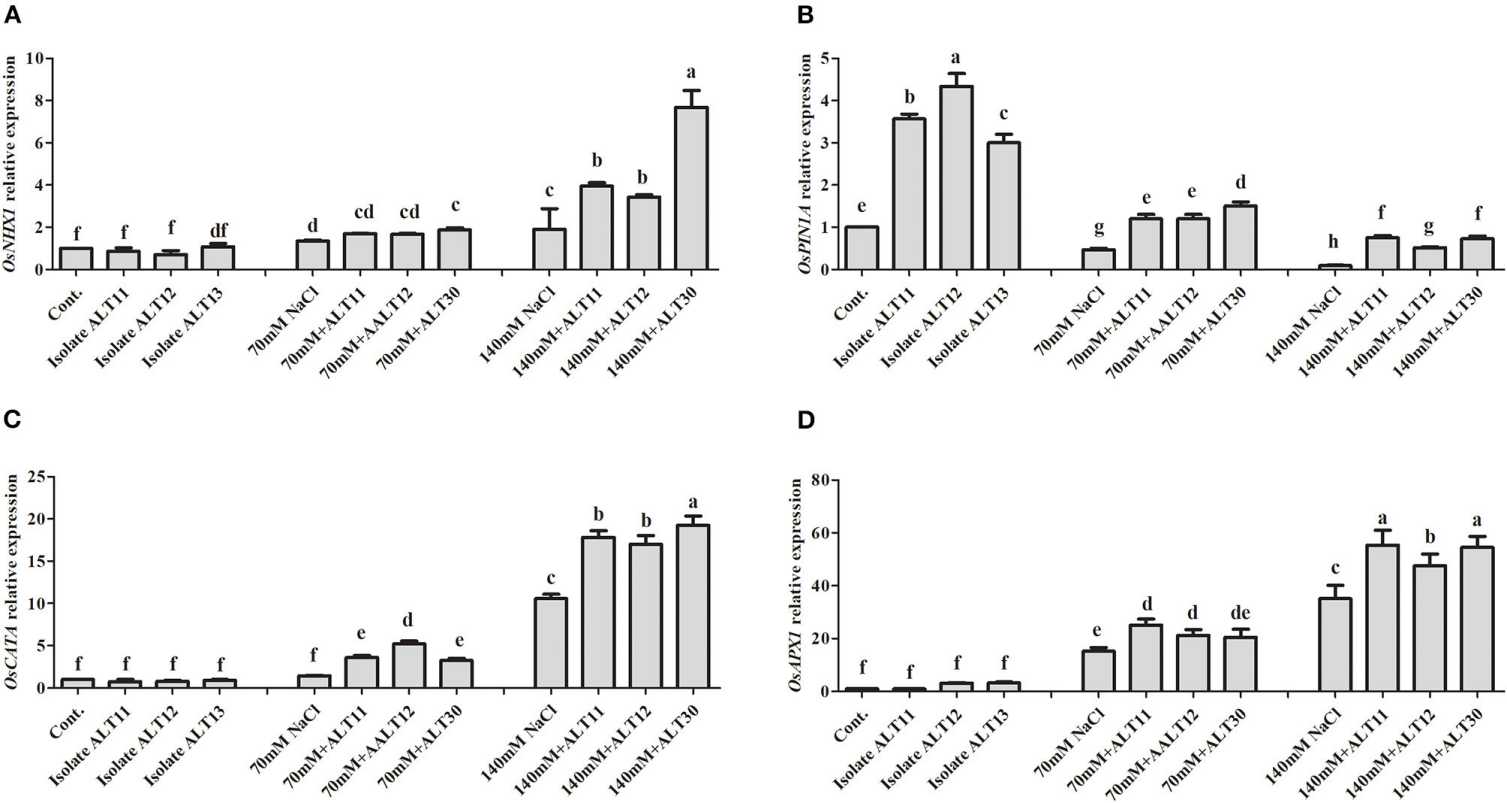
Gene expression in response to ALT11, ALT12, and ALT30 under normal conditions and NaCl stress. Relative expression of **(A)** *OsNHX1*, **(B)** *OsPIN1A*, **(C)** *OsCATA*, and **(D)** *OsAPX1* genes under normal conditions and NaCl stress (70 and 140 mM) calculated using actin gene expression. Different letters indicate significant differences between the mean values of three replicates ± standard deviation.

Gene expression was analyzed using an RT-PCR of rice under NaCl stress with and without inoculated bacterial isolates (Figure 7). The *OsNHX1* gene expression results showed that increasing salinity stress to 70 and 140 mM caused a higher expression of *OsNHX1* (0.9 and 8.5-fold, respectively) in rice plants (Figure 7A). However, decreases in the expression of *OsNHX1* (0.3 and 4.1-fold) were observed in rice plants inoculated with bacterial isolates under NaCl stress (Figure 7A). Similarly, *OsPIN1A* gene expression revealed that *OsPIN1A* was downregulated in rice plants under 70 and 140 mm NaCl stress compared with the control rice plants, whereas there was a higher expression of genes in rice plants inoculated with bacterial isolates than in rice plants treated only with NaCl (Figure 7B). Under normal conditions, there were no significant differences in *OsCATA*. However, rice plants treated with 70 and 140 mM NaCl showed enhanced *OsCATA* expression (Figure 7C). The bacterial isolates in inoculated rice plants augmented salinity stress through an antioxidant defense system and enhanced *OsCATA* expression under NaCl stress (Figure 7C). The *OsAPX1* gene was highly expressed (15–39-fold) under 70 and 140 mM NaCl stress. However, there was a higher expression of *OsAPX1* at 70 mM (7-fold) and 140 mM (17-fold) in bacterial-inoculated rice plants than in rice plants treated only with NaCl (Figure 7D).

## Discussion

Abiotic and biotic environmental stresses are among the most limiting factors to plant growth and productivity (Karlidag et al., 2010). Among these factors, salt stress is abiotic stress that negatively affects plant growth and crop productivity (Santos et al., 2018). High salinity induces numerous disorders in seed germination, growth, and yield (Yildirim et al., 2008). Salinity stress reduces plant growth due to an increase in Na uptake, increase in endogenous ABA content, excess ROS generation, and a reduction in photosynthetic rate and K uptake (Ansari et al., 2019). However, the interactions of PGPB reduce the extent of poor growth and help plants to survive in adverse saline conditions (Shukla et al., 2012). Our results suggest that rice plants inoculated with isolates ALT11, ALT12, and ALT30 showed better growth and higher tolerance to 70 and 140 mM NaCl stress (Figure 2, Table 1). Rice growth, biomass, and chlorophyll content were significantly decreased in plants only treated with NaCl, whereas there was an increase in the growth attributes and chlorophyll content of bacterial-inoculated rice plants under normal conditions and NaCl stress. This increase in the height and biomass of rice plants is probably due to the production of plant growth traits and tolerance to NaCl stress. Previous reports revealed that peanuts, radish, lettuce, strawberry, and wheat plants inoculated with bacterial isolates (*Brachybacterium* sp., *Staphylococcus* sp., *Bacillus* sp., and *Kocuria* sp.) showed higher growth rates and biomass (fresh/dry) than non-inoculated plants under salt stress (Yildirim et al., 2008, 2011; El-Tarabily and Youssef, 2010; Karlidag et al., 2010; Shukla et al., 2012; Desale et al., 2014; Liu et al., 2019). Amacher et al. (2000) revealed that PGPB reduces salinity stress in maize and wheat by ~50%. Similarly, leaf chlorophyll concentration is an indicator of salt tolerance and responds to increasing salinity stress (Habib et al., 2016; Shultana et al., 2020b). Salinity stress increases chlorophyllase activity, decreases chlorophyll synthesis, and destroys pigment proteins, decreasing chlorophyll pigment in alfalfa, lettuce, wheat, okra, and basil (Han and Lee, 2005; Bashan et al., 2006; Heidari and Golpayegani, 2012; Habib et al., 2016; Ansari et al., 2019). Our results showed that isolate inoculation of rice plants enhanced chlorophyll pigments and mitigated the harmful effects of salinity stress (Table 2). This increase in chlorophyll content in rice plants might be due to the beneficial effect of the inoculated isolates. Similarly, higher chlorophyll content was also reported for PGPB-inoculated salt-stressed rice, cucumber, and okra plants than for non-inoculated plants (Bal et al., 2013; Kang et al., 2014; Habib et al., 2016). Additionally, salinity stress affected carotenoid content, which plays an important role in the prevention of several degenerative stresses in plants (Babu et al., 2011). Other studies have shown that higher chlorophyll and photosynthetic contents in inoculated plants were related to higher absorption of iron and Mg (Hosseinzadah et al., 2011; Ansari et al., 2019). Mg is a macronutrient, and its absence adversely affects several functions in plants, including photosynthesis (Farhat et al., 2016). In plants, Mg is the most metabolically active mineral, with the highest concentration in chloroplasts (Waters, 2011). Mg shares a central atom in the tetrapyrrole ring of chlorophyll a and b molecules, which are considered essential pigments for photosynthetic light absorption (Waters, 2011; Tränkner et al., 2018). A decrease in Mg was found to adversely affect the photosynthesis rate (Hermans et al., 2005; Farhat et al., 2016). The ICP-MS analysis results for Mg showed that salinity stress (70 and 140 mM) decreased the level of Mg in rice plants compared with the control rice plants (Figure 6C). However, an increase in Mg content was observed in the bacterial-inoculated salt-stressed rice plant. Thus, a decrease in Mg content may contribute to a decrease in chlorophyll and photosynthetic pigments (Gomes et al., 2011). Previous studies also support our findings that plants subjected to salinity stress show a decrease in the Mg concentration (Hu and Schmidhalter, 2005; Jampeetong and Brix, 2009).

PGPB are a group of free-living microorganisms that colonize the roots of plants and play a beneficial role in various stages of plant growth, either directly, by increasing IAA, siderophore, and HCN, or indirectly, by inducing systemic resistance (Yildirim et al., 2008). EPS-producing PGPB play a significant role in alleviating salinity stress (Kumar et al., 2020), as EPS contains high molecular lipopolysaccharide proteins and polysaccharide lipids and binds with cations, such as Na, develop a soil sheath around the plant roots, and decrease the quantity of Na ions available for plant uptake (Shin et al., 2016; Kumar et al., 2020). EPS also helps in the establishment of plant–microbe interactions by providing a microenvironment in which microbes can survive under stress conditions, enhancing water retention, and regulating the diffusion of organic carbon sources (Kumar et al., 2020). Siderophores are a class of small-molecule compounds, which are highly specific for chelating Fe^3+^. Many PGPB acquire iron by producing siderophore and reduce the inhibitory effect of salinity stress by increasing photosynthesis and the chlorophyll content of plants (Ferreira et al., 2019; Liu et al., 2019; Kumar et al., 2020). Furthermore, several authors reported that siderophore-producing bacteria represent a promising alternative to chemical fertilizers due to tackling salt stress effects by enhancing the growth and biomass of soybean (El-Esawi et al., 2018), peanut (Paulucci et al., 2015), pepper (Wang et al., 2018), and wheat (Masalha et al., 2000). Moreover, laboratory assays conducted by Grobelak and Hiller (2017) and Shameer and Prasad (2018) reported an increase in plant growth as a result of inoculation with siderophore-producing bacteria, which constitutes direct evidence of the role of microbial siderophores in enhancing plant development. Additionally, phosphate is also important for plant growth. Phosphate-solubilizing halotolerant PGPB provides an opportunity to enhance P availability by solubilizing insoluble phosphate via various mechanisms, including secreting low molecular weight organic acids (Sharma et al., 2013; Etesami, 2018). The production of organic acids is the main mechanism used by microbes to mineralize inorganic P (Rodriguez et al., 2004). The organic acids produced by PGPR include malic, acetic, citric, oxalic, lactic, formic, gluconic, and 2-keto-gluconic acids (Vázquez et al., 2000; Vessey, 2003). Phosphate-solubilizing organic acid-producing microbes are considered important members of PGPR, and their application as biofertilizers has been shown to improve the growth of crops plants by increasing P availability to plants, reduce fertilizers inputs, and fulfill the metabolic demands of plant (Vessey, 2003; Khan et al., 2007; Panhwar et al., 2011). In salt-affected soil, the inoculation of phosphate-solubilizing halotolerant PGPB improves plant growth and suppresses the adverse effect of salt (Giri et al., 2004; Etesami and Beattie, 2018). Similarly, *Bacillus aryabhattai* and *Bacillus megaterium* are efficient halotolerant P-solubilizing microbes under saline conditions (Chookietwattana and Maneewan, 2012; Bhattacharyya et al., 2017). The production of IAA is a relatively common trait of most salt-tolerant PGPBs, and it increases the fitness of plant growth in salt-affected soils. IAA-producing, salt-tolerant *Planococcus rifietoensis, Brachybacterium*, and *Haererohalobacter* enhance the growth and yields of wheat and peanut plants under salinity stress (Shukla et al., 2012; Desale et al., 2014; Zhou et al., 2015). In this study, all selected isolates produced IAA, whereas isolate ALT11 produced the highest amount of IAA (Figure 1A). Furthermore, PIN protein plays an important role in facilitating auxin efflux, and salinity stress downregulates the expression of PIN genes. In this study, a decrease in *OsPIN1A* was observed in salt-stressed rice plants. However, in bacterial-inoculated rice plants, there was an increase in the expression of *OsPIN1A* genes (Figure 7B). Similar results were also reported by Fu et al. (2019) who found that salinity stress leads to a reduction in root meristem size through the downregulation of PIN genes, thereby reducing plant auxin levels. The increase in PIN gene expression in rice plants might be due to the production of IAA and the halotolerance of the selected isolates ALT11, ALT12, and ALT30.

The content of Na and K in rice was significantly affected under treatments of 70 and 140 mM NaCl. In this study, rice plants exposed to salinity stress had increased Na ion concentrations in their shoots (Figure 6A). This increase in Na content was negatively correlated with rice growth (root/shoot), biomass (fresh/dry), and chlorophyll content. Arora et al. (2010) reported that, under salinity stress, bacteria can bind Na ions through the secretion of EPS, which reduces Na toxicity in soil. A higher population of EPS-producing bacteria in the root rhizosphere reduces the concentration of Na availability for uptake and alleviates the NaCl effect on plants (Shultana et al., 2020b). In current observations, a decrease in Na content in bacterial-inoculated rice plants might be due to their EPS-producing activity (Supplementary Figure 1B). K is an inorganic solute, and it contributes to maintaining osmotic pressure, ionic strength, and osmotic adjustment in plants under normal and salt stress conditions (Santos et al., 2018). In general, the antagonism between Na and K ions promotes an increase in Na ions and a decrease in K content. Additionally, maize plants treated with an *Azotobacter* strain have enhanced K absorption and decreased Na content under salinity stress (Rojas-Tapias et al., 2012). According to Hauser and Horie (2010) and Shukla et al. (2012), salt-tolerant plants are able to maintain favorable K homeostasis during periods of salinity stress. The results of our study showed that plants inoculated with an isolate had enhanced K content and decreased Na content under salinity stress (Figure 6B). This increase in K content in rice plants was positively correlated with rice growth and biomass. Recent studies of plants inoculated with different PGPB demonstrate that microbes decrease Na content under salinity stress (Volkov, 2015; Ansari et al., 2019). Additionally, Etesami and Beattie (2018) and Shukla et al. (2012) showed that halotolerant bacteria could enhance K absorption in plants under salinity stress. Further, under higher salinity stress, the NHX family members were differentially expressed in bacterial-inoculated and non-inoculated rice plants. In rice, five *OsNHX1* genes have been identified (Fukuda et al., 2011; Zhang et al., 2018) and play an important role in the compartmentalization of Na and K in the cytoplasm (Fukuda et al., 2011). Fukuda et al. (2011) and Fukuda et al. (2004) reported that the overexpression of *OsNHX1* improves the salinity tolerance of transgenic rice. Similar results were observed in rice plants inoculated with all three bacterial strains (Figure 7A). Chen et al. (2007) reported that overexpression of *OsNHX1* enhanced growth in maize plants in 200 mM of NaCl stress.

The protective role of ABA is pivotal in plant growth as it promotes stomatal closure to minimize water loss and mediate stress damage through the activation of stress response genes, which collectively increase plant stress tolerance (Kang et al., 2014). It has been widely described that the ABA content of plants increases under salinity stress. However, we found a significant decrease in ABA content in the presence of bacterial-inoculated plants compared with non-inoculated salt-stressed plants. There are several studies that narrate the same finding of low ABA content in PGPB-inoculated plants under abiotic stress, including salinity stress (Kang et al., 2014; Jan et al., 2019; Kim et al., 2020). Similarly, various bacteria have been reported with both increased (Salomon et al., 2014) and decreased ABA concentration (Zhang et al., 2008) in plants. In tomato plants, the ABA-utilizing bacteria was found to decrease ABA concentration (Belimov et al., 2014). However, some bacteria that cannot utilize ABA were also reported in maize, tomato, and pea plants to decrease ABA concentration by decreasing xylem and phloem ABA flows (Dodd et al., 2009; Jiang et al., 2012). These bacterial-mediated changes in ABA concentration may affect stomatal conductance to maintain photosynthesis (Salomon et al., 2014). Moreover, the mechanisms of bacterial moderation of plant ABA concentrations may differ depending on the traits of a particular strain. Identifying bacterial genes and enzymes responsible for ABA catabolism, as well as obtaining knockout mutants of the bacteria unable to utilize this compound, would further evaluate the physiological and ecological significance of this bacterial trait. However, SA induces systemic acquired resistance and improves plant growth under stress (Kang et al., 2014). In dicot plants, such as *Nicotiana* and *Arabidopsis*, SA exists at low levels, whereas in rice, the SA contents are higher under normal growth conditions (De Vleesschauwer et al., 2013), as various physiological and biochemical responses are regulated. ROS, such as SOAs and hydrogen peroxide, are normally produced at high rates and cause oxidative damages to the cell structure during salt stress conditions (Sharma et al., 2012; Rodrigues et al., 2013). However, a defensive system, called the antioxidant enzyme system, is also activated to remove the free radicals produced under stress conditions and maintain them at low levels (Habib et al., 2016). This system consists of several ROS-scavenging enzymes, such as POD, SOD, APX, CAT, which have the ability to mitigate abiotic stress, including salinity stress, remove free radicals, and avoid the toxicity of ROS produced in cells during stress (Santos et al., 2018). Improved control of the O_2_ level was observed in rice plants coinoculated with halotolerant isolate. However, O_2_ and H_2_O_2_ can attack and oxidize polyunsaturated fatty acids in the cell membrane, which results in an increase in lipid peroxidation (Pandey et al., 2017; Santos et al., 2018). In this study, rice plants coinoculated with all three bacterial isolates showed better control of lipid peroxidation under normal and salt stress conditions (Figure 4D). Similar results were also observed in cowpea plants coinoculated with *Bradyrhizobium, Paenibacillus durus*, and *Paenibacillus graminis* (Egamberdieva et al., 2013; Santos et al., 2018). GSH occurs in reduced GSH and oxidized forms and protects the cell membrane against ROS damage (Sytar et al., 2013; Santos et al., 2018). CAT and SOD are metalloproteins with an important role in controlling ROS levels (Santos et al., 2018). SOD catabolizes O_2_ to H_2_O_2_, which is further catabolized by CAT, and excess H_2_O_2_ is removed to produce H_2_O and O_2_ under salinity stress (Conklin and Barth, 2004; Jamil et al., 2011; Sytar et al., 2013; Pandey et al., 2017). In this study, rice plants inoculated with the isolates ALT11, ALT12, and ALT30 had increased SOD and CAT activities under salinity stress, whereas there was a decrease in H_2_O_2_ and lipid peroxidation. This result provides evidence of the beneficial effect of microbes on the positive balance of antioxidative enzymes that detoxify ROS metabolism (Santos et al., 2018). Similarly, high gene expressions of *OsCATA* and *OsAPX1* were observed in bacterial-inoculated rice plants under salinity stress. CAT and APX enzymes are encoded by different gene families. In rice, enzymatic antioxidants such as CAT and APX help convert excess H_2_O_2_ to H_2_ and O_2_ (Rossatto et al., 2017; Peng et al., 2018). Our results also support the findings of studies by Rossatto et al. (2017) and Kim et al. (2007), who report a higher expression of *OsAPX* and *OsCAT* genes under salinity stress. The increase in the expression of these genes during stress conditions is correlated with an increase in antioxidant enzymatic activities.

## Conclusions

This study suggests that the tested isolates (ALT11, ALT12, and ALT30) play a pivotal role in mitigating NaCl tolerance (70 and 140 mM) in rice. Inoculation with halotolerant bacterial isolates showed improved growth, biomass, and chlorophyll content by altering endogenous phytohormones, activating an antioxidant defense system, ion uptake, and regulating salt-related genes under normal and salinity stress in rice plants. Therefore, based on our findings, further studies should examine the genomics of our novel bacterial strains and conduct saline field trials to take this research to the farmer's fields for its practical application.

## Data Availability Statement

The original contributions presented in the study are included in the article/Supplementary Material, further inquiries can be directed to the corresponding author/s.

## Author Contributions

MAK conducted the experiments. MH and SA helped in the writing of the manuscript. MAK and S-MK conducted hormonal and antioxidant analyses. MK and BW-Y conducted RT-PCR analysis. I-JL designed, supervised, and financed the research. All authors have read and agreed to its content and that the manuscript conforms to the journal's policies.

## Conflict of Interest

The authors declare that the research was conducted in the absence of any commercial or financial relationships that could be construed as a potential conflict of interest.
