# Supplementary material for: Rhizospheric Bacillus spp. Rescues Plant Growth Under Salinity Stress via Regulating Gene Expression, Endogenous Hormones, and Antioxidant System of Oryza sativa L
Source: Front Plant Sci. 2021 Jun 11;12:665590. doi: 10.3389/fpls.2021.665590 (PMC8226221; doi:10.3389/fpls.2021.665590)

**Fig. S1:** Assessment of bacterial isolates for beneficial plant-growth promoting activities. (A) Luria–Bertani media plates (B) Exopolysaccharide (EPS) activity on Congo red medium, (C) Chromeazurol “S” agar plates for siderophore production, (D) Phosphate solubilization, and (E) Salkowski reagent assay for indole-3-acetic acid production.


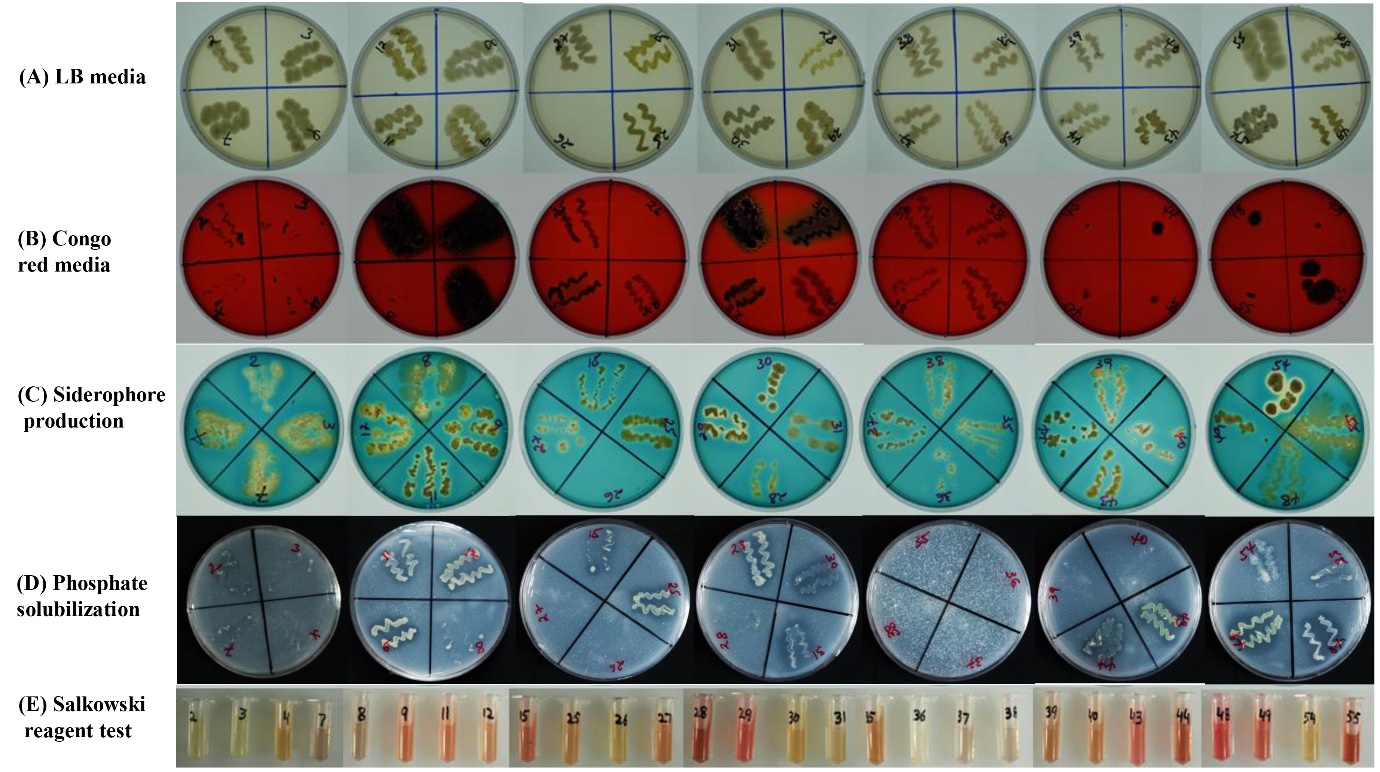


**Fig. S2:** Growth of multiple plant-growth promoting traits produced by rhizospheric bacteria. Plant-growth promoting rhizobacteria were grown in Luria–Bertani media supplement with 0, 50, 100, and 150 mM NaCl for 48 h, and the growth was examined using a spectrophotometer at 600 nm. Each data point is the mean of three replications.


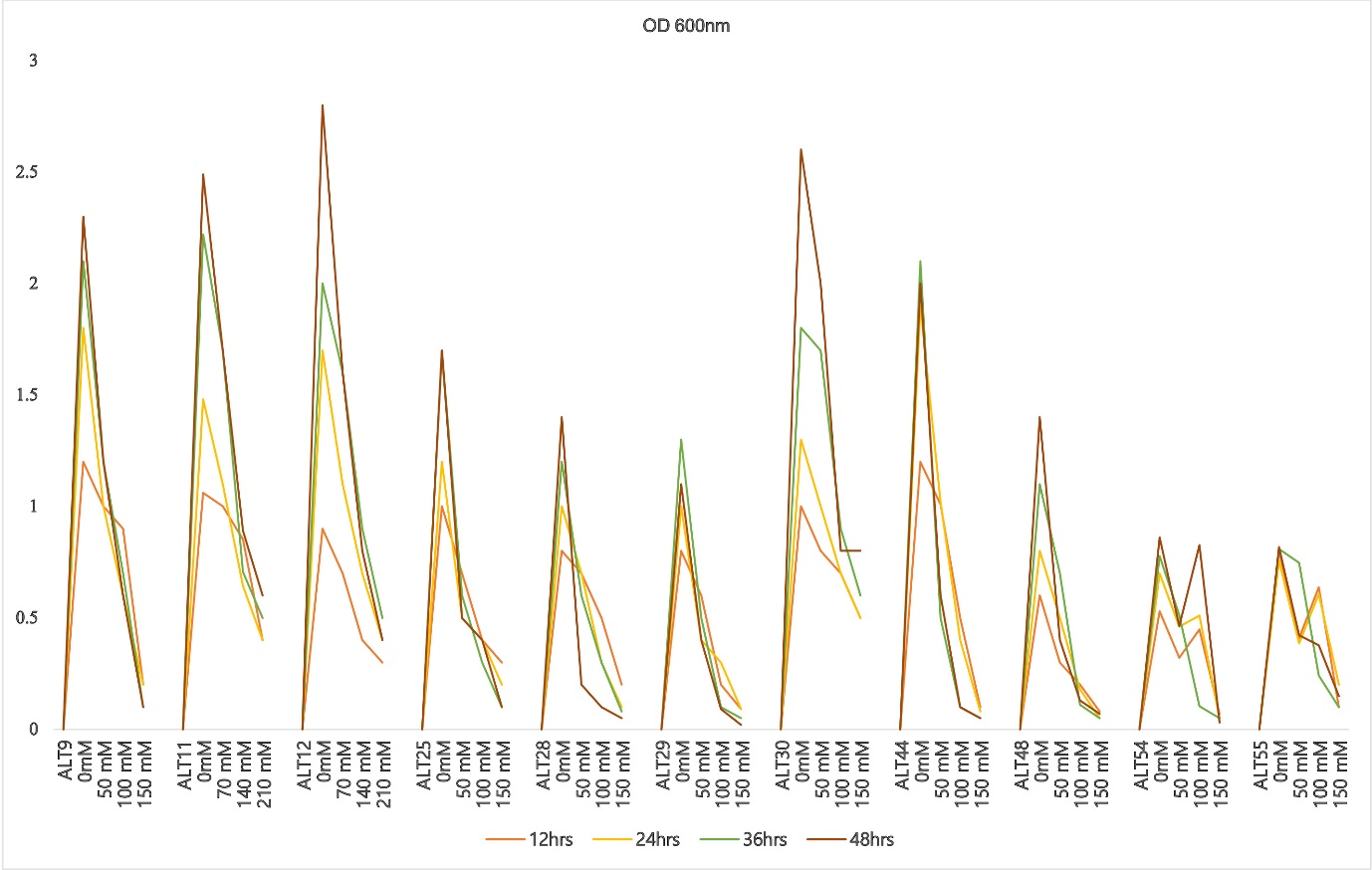


**Fig. S3:** Phylogenetic tree of ALT11, ALT12, and ALT30 constructed using 16S rRNA sequences using neighbor-joining and maximum-likelihood methods.


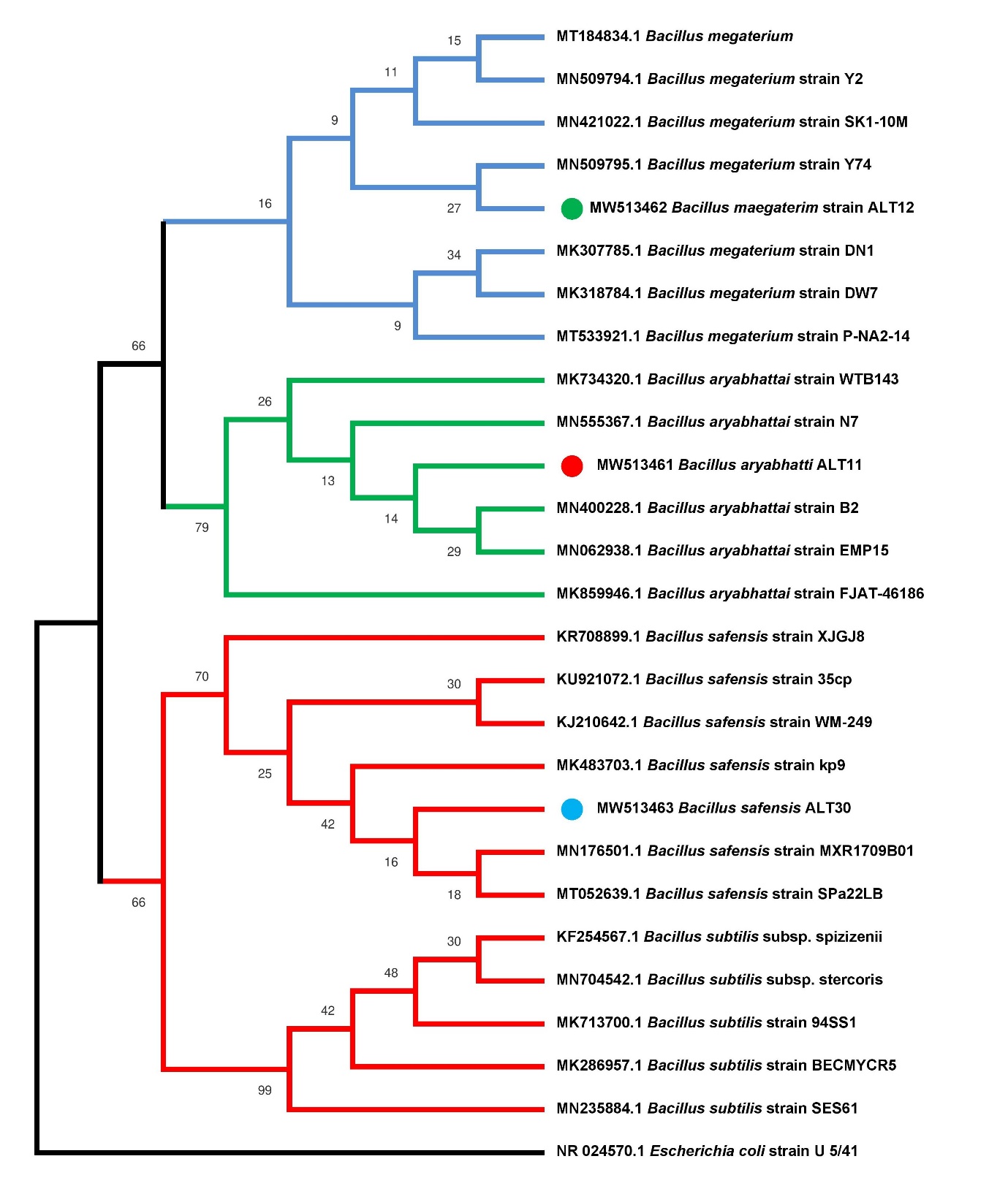

Supplement: Supplementary file 1 [file Data_Sheet_1.DOCX]
