# Supplementary material for: Rhizospheric Bacillus spp. Rescues Plant Growth Under Salinity Stress via Regulating Gene Expression, Endogenous Hormones, and Antioxidant System of Oryza sativa L
Source: Front Plant Sci. 2021 Jun 11;12:665590. doi: 10.3389/fpls.2021.665590 (PMC8226221; doi:10.3389/fpls.2021.665590)
Supplement: Supplementary file 2 [file Data_Sheet_2.docx]

**S. Table 1: The primers used for real-time PCR.**

| Gene | Primer sequences |
| --- | --- |
| *Os*NH*X*1 | Forward: GCTAGATTTGAGCGGCATTC  Reverse: GAAGGCTCAGAGGTGACAGG |
| *OsAPX1* | Forward: CCAAGGGTTCTGACCACCTA  Reverse: CAAGGTCCCTCAAAACCAGA |
| *OsCATA* | Forward: CGGATAGACAGGAGAGGTTCA  Reverse: AATCTTCACCCCCAACGACT |
| *OSPIN1A* | Forward: TCATCTGGTCGCTCGTCTGC  Reverse: CGAACGTCGCCACCTTGTTC |
| *OsACT1* | Forward: GTATCCATGAGACTACATACAACT  Reverse: TACTCAGCCTTGGCAATCCACA |

**S. Table 2.** Isolation of rhizospheric bacteria having individual or

multiple plant growth promoting characteristics.

| NO | EPS production | Siderophore production | Phosphate solubilization | IAA production |
| --- | --- | --- | --- | --- |
| ALT1 | - | - | - | - |
| ALT2 | - | - | - | - |
| ALT3 | - | + | - | - |
| ALT4 | - | + | - | + |
| ALT5 | - | - | - | - |
| ALT6 | - | - | - | - |
| ALT7 | - | + | - | - |
| ALT8 | - | - | - | - |
| ALT9 | + | + | + | + |
| ALT10 | + | - | - | - |
| ALT11 | + | + | + | + |
| ALT12 | - | + | - | + |
| ALT13 | - | - | - | - |
| ALT14 | - | - | - | - |
| ALT15 | + |  |  | + |
| ALT16 | - | - | - | - |
| ALT17 | - | - | - | - |
| ALT18 | - | - | - | - |
| ALT19 | - | - | - | - |
| ALT20 | - | - | - | - |
| ALT21 | - | - | - | - |
| ALT22 | - | - | - | - |
| ALT23 | - | - | - | - |
| ALT24 | - | - | - | - |
| ALT25 | + |  | + | + |
| ALT26 | - | - | - | + |
| ALT27 | - | + | - | + |
| ALT28 | - | - | - | + |
| ALT29 | + | + | + | + |
| ALT30 | + | + | + | + |
| ALT31 | + | - | + | - |
| ALT32 | - | - | - | - |
| ALT33 | - | - | - | - |
| ALT34 | - | - | - | - |
| ALT35 | - | - | - | + |
| ALT36 | - | - | - | - |
| ALT37 | - | - | - | - |
| ALT38 | - | - | - | - |
| ALT39 | - | + | - | + |
| ALT40 | - | + | - | + |
| ALT41 | - | - | - | - |
| ALT42 | - | - | - | - |
| ALT43 | + | + | + | + |
| ALT44 | + | + | + | + |
| ALT45 | - | - | - | - |
| ALT46 | - | - | - | - |
| ALT47 | - | - | - | - |
| ALT48 | + | - | + | + |
| ALT49 | + | - | + | + |
| ALT50 | - | - | - | - |
| ALT51 | - | - | - | - |
| ALT52 | - | - | - | - |
| ALT53 | - | - | - | - |
| ALT54 | - | + | - | + |
| ALT55 | - | - | - | + |
